# P-349. Identification and Control of a *Candida auris* Outbreak in an Acute Spinal Cord Injury Unit at a Veterans Affairs Medical Center

**DOI:** 10.1093/ofid/ofae631.550

**Published:** 2025-01-29

**Authors:** Matthew M Hitchcock, Michele S Fleming, Shaunta Poe, Shelley Knowlson, Melanie Christian, Angela Eckert, Emily Hill, John D Markley

**Affiliations:** Central Virginia VA Health Care System, Richmond, Virginia; Central Virginia VA Health Care System, Richmond, Virginia; Central Virginia VA Health Care System, Richmond, Virginia; Central Virginia VA Health Care System, Richmond, Virginia; Central VIrginia VA Health Care System, Richmond, Virginia; Central Virginia VA Health Care System, Richmond, Virginia; Central Virginia VA Health Care System, Richmond, Virginia; Central Virginia VA Health Care System, VCU Medical Center, Richmond, Virginia

## Abstract

**Background:**

*Candida auris* is an emerging pathogen that spreads easily in healthcare settings and can cause severe infections. In Dec. 2022, *C. auris* was identified at the Richmond VA Medical Center (VAMC) in an acute care spinal cord injury unit (SCIU). The SCIU is a regional SCI center in a 300-bed VAMC that provides medical and rehabilitation services to patients from multiple states.

**Figure 1.**
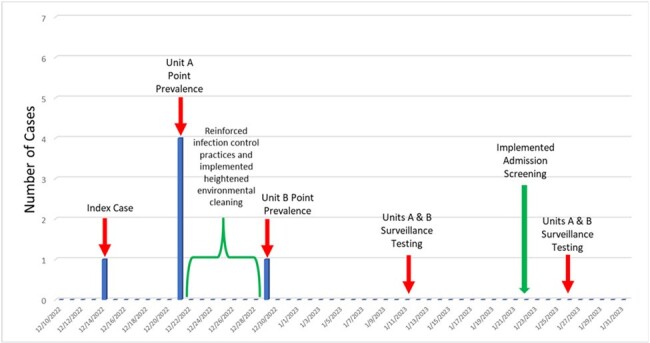
Point prevalence testing results and timeline of interventions during the outbreak investigation.

**Methods:**

*C. auris* was identified in 1 urine culture from a SCIU patient, triggering an outbreak investigation. The Infection Prevention (IP) team performed point prevalence (PP) testing via superficial swabs of axilla and groin sent for polymerase chain reaction (PCR) testing. Initial screening included 31 unique patients in 2 units, A (n=16) and B (n=15). Admission testing was also initiated. Patients were isolated until tests resulted. Surveillance PP testing was performed biweekly and ceased after consecutive rounds of negative results. The IP team evaluated infection control practices in the SCIU that could contribute to transmission.

**Results:**

The initial screening of 31 patients yielded 5 positives (16%; Figure 1). Overall, 92 PCR tests were completed on 51 unique patients for a positivity rate of 9.8% (n=5). Positive results occurred only during the initial testing. Positive patients were admitted for a median of 36 days (range: 16-412). No *C. auris* was isolated in culture and no invasive infections developed. Records and interviews revealed complex movement between healthcare facilities for the colonized patients. Multiple lapses in infection control practice were found. Admission testing with repeat testing at 90 days was implemented longitudinally, along with enhanced cleaning measures and reinforcement of transmission-based precautions (Figure 1). No new positive results were found over the subsequent 9 months.

**Conclusion:**

PP testing for *C. auris* in an SCIU identified 5 colonized patients. As no isolates were recovered beyond the index case, IP could not determine whether this outbreak was due to intra-unit transmission versus multiple introductions. Improvements in infection control practices, along with implementation of surveillance testing, contributed to control of the outbreak. The SCIU remains vulnerable to *C. auris* due to the complex, multidisciplinary care needs of the patient population.

**Disclosures:**

**All Authors**: No reported disclosures

